# Face Orientation and Motion Differently Affect the Deployment of Visual Attention in Newborns and 4-Month-Old Infants

**DOI:** 10.1371/journal.pone.0136965

**Published:** 2015-09-14

**Authors:** Eloisa Valenza, Yumiko Otsuka, Hermann Bulf, Hiroko Ichikawa, So Kanazawa, Masami K. Yamaguchi

**Affiliations:** 1 Dipartimento di Psicologia dello Sviluppo e Socializzazione, Università degli Studi di Padova, Via Venezia 8, 35131, Padova, Italy; 2 Interdepartmental Center for Cognitive Science (CISC), Università di Padova, Via Venezia 8, 35131, Padova; 3 School of Psychology, UNSW Australia, Sydney, NSW, 2052, Australia; 4 Department of Psychology, University of Milano-Bicocca, Piazza Ateneo Nuovo 1, 20126, Milano, Italy; 5 Milan Center of Neuroscience (NeuroMI), Milan, Italy; 6 Department of Psychology, Chuo University, Hachioji-city, Tokyo, 192–0393, Japan; 7 Japan Society for the Promotion of Science, Chiyoda-ku, Tokyo, 102–0083, Japan; 8 Department of Psychology, Japan Women’s University, Kawasaki, Kanagawa, 214–8565, Japan; Vanderbilt University, UNITED STATES

## Abstract

Orienting visual attention allows us to properly select relevant visual information from a noisy environment. Despite extensive investigation of the orienting of visual attention in infancy, it is unknown whether and how stimulus characteristics modulate the deployment of attention from birth to 4 months of age, a period in which the efficiency in orienting of attention improves dramatically. The aim of the present study was to compare 4-month-old infants’ and newborns’ ability to orient attention from central to peripheral stimuli that have the same or different attributes. In Experiment 1, all the stimuli were dynamic and the only attribute of the central and peripheral stimuli to be manipulated was face orientation. In Experiment 2, both face orientation and motion of the central and peripheral stimuli were contrasted. The number of valid trials and saccadic latency were measured at both ages. Our results demonstrated that the deployment of attention is mainly influenced by motion at birth, while it is also influenced by face orientation at 4-month of age. These findings provide insight into the development of the orienting visual attention in the first few months of life and suggest that maturation may be not the only factor that determines the developmental change in orienting visual attention from birth to 4 months.

## Introduction

Research suggests that attention may be conceptualized as a collection of dissociable functions or components, each with a dedicated underlying central nervous system pathway or structure [1, 2]. Orienting visual attention is one such function and the topic of this paper. Efficiency of orienting visual attention is crucial to exploring the environment for further processing and learning [3]. It undergoes dramatic developments in the first year of life, including acquisition of the capacity to disengage attention and look away from salient or captivating stimuli impinging on the fovea [4, 5]. Disengagement from fixation is a prerequisite for initiating a saccade to a new target and has been studied in controlled laboratory situations using the gap-overlap paradigm. This paradigm measures the flexibility in attentional switching in response to changes in the visual environment and may also be used to investigate the disengagement of attention during infancy [4, 6–11]. Infants’ gaze is first drawn to a visual stimulus on the midline and then a stimulus is presented in the visual periphery. This typically draws the eyes away from the midline stimulus and towards the peripheral stimulus. If a temporal gap is introduced between the disappearance of the midline stimulus and the appearance of the peripheral stimulus, the saccadic latency to reach the new target is reduced. This is presumably because disengagement is no longer necessary prior to shifting attention towards the peripheral stimulus. Conversely, if the midline stimulus remains present while the peripheral stimulus appears, the saccadic latency to reach the new target increases as disengagement of attention is now required prior to any shift away from the midline stimulus.

Earlier research has shown that when two stimuli are presented at the same time several factors may influence the orienting of attention from the central to the peripheral stimuli (i.e., overlap condition). For instance, disengagement is affected by the infant’s age, as it is well documented by the phenomenon of obligatory attention. Obligatory attention is the difficulty 1-2-month-old infants’ have in breaking gaze from the stimulus they are fixating [12]. Saccadic reaction time of younger infants are particularly impaired in the overlap condition relative to those of older infants [13, 14]. The frequency and speed of shifts of gaze to a target in the periphery when a stimulus remains present in the center increase substantially around 3–4 months of age [5, 15, 16]. By 4 months of age, infants are able to move their attention and gaze easily and rapidly, and staring behaviour becomes rare [9, 14, 16].

Orienting is affected also by stimulus attributes. Finlay and Ivinskies [17] demonstrated that a comparatively salient stimulus in the central visual field makes it more difficult for infants to disengage their gaze. For example, Hunnius and Geuze [9] recently investigated the efficiency of orienting attention from central to peripheral stimuli in ecological contexts using two different dynamic stimuli: a socially relevant stimulus and an abstract stimulus. The former consisted of a short video sequence of the face of the infant’s mother, and the latter consisted of a stimulus that matched the video of the mother in terms of dynamic characteristics, colour range, and luminance. These stimuli both appeared as central stimulus and as peripheral target. Infants were more likely to shift their gaze when the central stimulus was a face and the peripheral target was an abstract stimulus, while they were less likely and also slower to shift gaze in the opposite condition (i.e., abstract central, face peripheral). This effect was most marked between 10 and 18 weeks, whereas at 6 weeks of age, the infants rarely looked away from the central stimulus regardless of what it was or of what was presented in the periphery.

Taken together these results suggest that the efficiency of orienting attention from central to peripheral stimuli, specified in terms of the saccadic reaction time to reach the peripheral target, may be affected by the age of the viewer and by the characteristic of the central and the peripheral stimuli. While at birth disengagement is not yet completely efficient, by 4 months of age, infants are able to move their attention and gaze easily, rapidly and efficiently [9, 14, 16]. Starting from these findings it becomes very relevant to explore how the characteristics of the central and the peripheral stimulus influence the deployment of attention differentially at birth and at 4 months of age.

The aim of the present study was to compare the ability of 4-month-old infants and newborns to orient attention away from central to peripheral stimuli (i.e., overlap condition) in two experiments in which the saccade latency toward peripheral stimuli was recorded. In both experiments two attributes of the central and peripheral stimuli were manipulated: orientation of the face (upright vs. inverted) and motion (dynamic vs. static).

There are mainly two ways in which facial orientation affects face processing in young infants. First, facial orientation affect infants’ recognition of faces. It has long been known that faces are extremely difficult to recognize when seen upside down (i.e. face inversion effect). The face inversion effect refers to the greater impairment in performance for face recognition relative to object recognition due to stimulus inversion [18]. It has been assumed that the face inversion effect found in adults originate from the prolonged experience with upright face during the course of development. However, a number of studies have shown that the face inversion effect is present very early during development. These include a few studies reporting somewhat better face recognition for upright than inverted faces even at birth [19, 20] and a greater number of studies in infants over 4-months of age [21–24]. These findings suggest that infants’ early representation of faces contains information that is orientation-specific.

Second, and more relevant to the current study, upright faces are known to recruit attention from birth [25–26]. When presented with face-like and nonface-like patterns, newborns spontaneously look longer at, and orient more frequently toward, upright face-like configurations [26–27]. An upright face preference at birth has been demonstrated with both static and moving stimuli [28–31], and with both schematic and veridical images of faces [20–22]. Note, however, that at birth upright the face preference seems to depend on the existence of general biases that orient newborns’ attention toward certain structural properties that faces share with other visual stimuli [32–34]. One such structural properties is the so called “top-heavy” property, and it consists of a greater number of high contrast elements in the upper half of the configuration. At birth, top-heavy configurations made of geometrical patterns embedded in a rectangular area can elicit preference over the inverted versions of such configurations, analogous to the preference for upright faces. These findings suggest that newborns have only very crude representation about faces even if it is orientation-specific. However, a change in the determinants of infants’ face preference seems to occur in the following few months. At 3 months of age, upright face preference appears to become more selective to those perceptual characteristics that distinguish faces from other stimulus categories [35]. Moreover, by 3 months of age infants begin to show evidence of the formation of face prototypes [36]. Overall, this pattern of results is consistent with an experience-expectant perspective on face processing. The experience-expectant perspective emphasize the relevance of both general biases and exposure to certain experiences in driving functional specialization of face processing during the first months of life [37–41]. There is also evidence showing that facial orientation influences the pattern of eye movement by 4-month-olds while exploring faces [42]. During the presentation of upright faces, 4-month-old infants spent more time exploring the internal features of faces. Moreover, they alternated their gaze as frequently between the face’s internal features as between external and internal features. However, the exploration and the gaze shift between the internal features decreased during the presentation of inverted faces, suggesting that the attentional mechanisms involved in infants’ face processing are different for upright and inverted faces. Accordingly with these results, Valenza, Franchin, and Bulf [43] have recently showed that upright and inverted faces differently affect the deployment of attention in 8-month-old infants. Using a Posner-like cuing task, they found that infants spent more time in shifting attention between two inverted faces, but not between two upright faces.

In addition to face orientation, dynamic properties of stimuli are also known to attract infants’ attention from birth. It is a commonplace observation that newborns readily attend to moving visual stimuli and that they show a visual preference for dynamic stimuli, such as flickering or flashing stimuli [44–46]. In the presence of a central stimulus, newborns localize peripheral stimuli at greater eccentricities when the peripheral stimulus is flashing than when it is not flashing [47]. The frequency of detection of a target in the periphery increased between 2 and 10 weeks of age when the central stimulus was static and the peripheral target was moving, but not when the central stimulus was moving and the peripheral target was static [48]. Similarly a flickering peripheral stimulus affects the probability of, and latency to, move fixation from a central location to the peripheral stimulus [49]. These findings show that attention to a focal dynamic stimulus attenuates detection of a peripheral stimulus while flickering or movement in the peripheral stimulus increases the likelihood of its detection and decreases the latency of the associated eye movement. Previous studies have shown that sensitivity to various motion information undergoes considerable development in the first few months after birth [50]. However, there is evidence showing that motion is a rich source of information from birth. Newborns preferentially look at point-light display depicting biological movement over non-biological movement, demonstrating the detection of the movement of biologically relevant signals [51]. There are also studies reporting facilitative effect of motion on newborns’ and infants’ face recognition [52–53], while others have shown that facial movements may distract newborns’ attention and consequently disrupt their recognition of dynamic talking face [54–55].

When considered together both facial orientation and motion trigger attention and it is a relevant source of information even from birth. In order to find out how these attributes modulate the orienting of attention during the course of development, we conducted the following two experiments. First, we manipulated the orientation of the central and peripheral face using faces in motion (Experiment 1). Second, we contrasted face orientation attribute and motion attribute of the stimuli between the central and peripheral stimuli (Experiment 2).

## Experiment 1

Experiment 1 examined the possibility that the orientation of the central and peripheral face stimuli may differently affect the deployment of visual attention in 4-month-old (Experiment 1a) and in newborn infants (Experiment 1b). In Experiment 1, all facial stimuli were shown in dynamic condition, and only their orientation was manipulated. Given that only face orientation is manipulated in Experiment 1, it might be expected that this attribute would affect orienting in a similar way for both the age groups. That is, it might be expected that, for both age groups, the latency of orienting will be longer when the central face is upright compared to when it is inverted and also when the peripheral face is inverted compared to when it is upright. Alternatively, motion per sè is an effective producer of newborns’ visual attention and effect of motion may dominate over the effect of face orientation at birth. Given that both the central and the peripheral stimuli are dynamic, dynamic property of stimuli may overwhelm the effect of facial orientation for newborns who has only crude representation about faces. Then, face orientation might only affect orienting in the 4-month-old infants.

### Ethics statement

**Fig 1:**
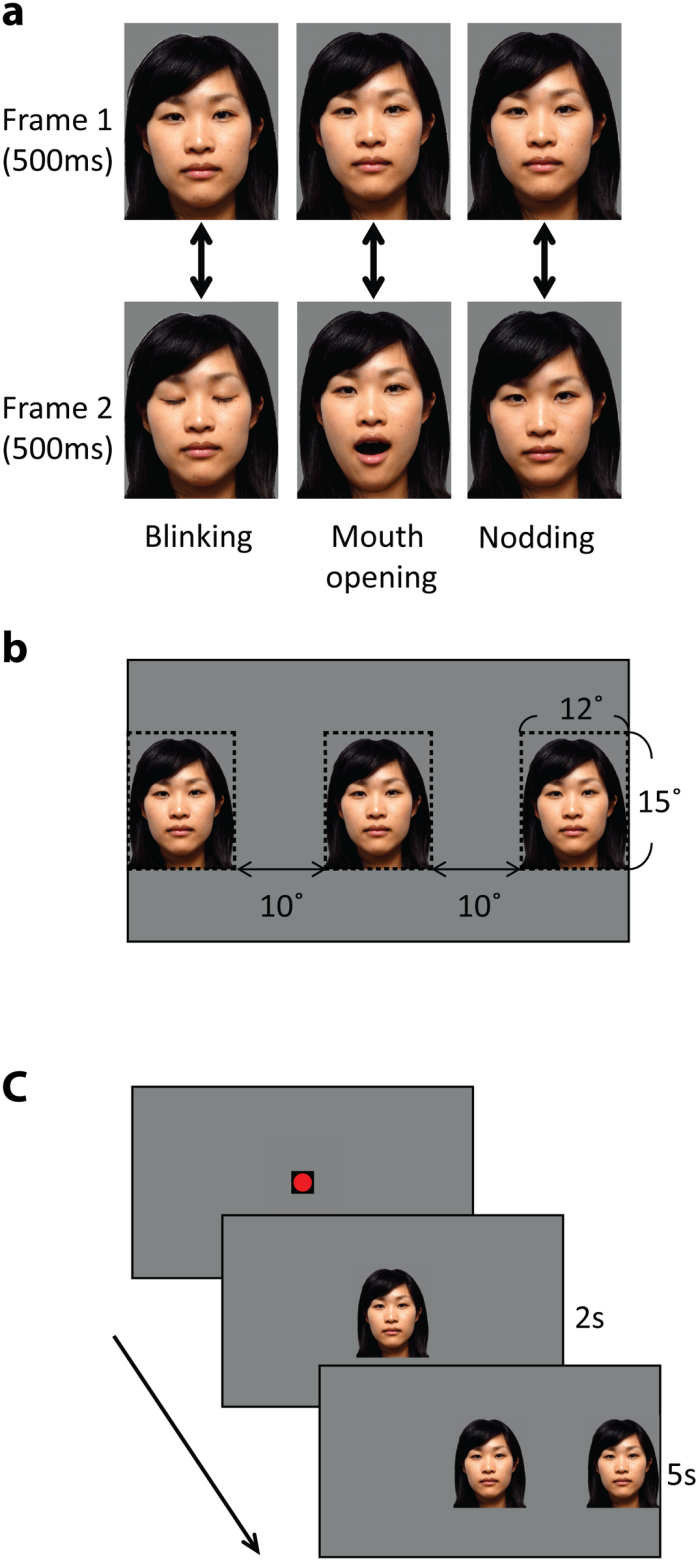
Stimuli used in Experiment 1. (a) Example of dynamic stimuli showing still from the three motion types in upright orientation. Dynamic impression was created by showing the frame 1 and the frame 2 alternately at 1 Hz. Frame 1 was shown at the onset of the stimuli, and was also used for static stimuli. (b) Schematic representation of central and peripheral stimulus position. The dotted area around the middle face represents the central AOI, while those around the left and right faces represent the peripheral AOI. (c) Schematic representation of stimulus sequence showing a trial where the peripheral stimulus appears on the right. As soon as infants fixate on the fixation point, experimenter started the trial. The central face remained on the screen for 2seconds before presentation of peripheral face which ramined on the screen together with the central face for another 5 seconds for 4-month-olds or 10 seconds for newborns.

Participants of this study were tested after their parents had provided written informed consent. The protocol was carried out in accordance with the ethical standards of the Declaration of Helsinki (BMJ 1991; 302: 1194) and approved by the Chuo University Human Research Ethics Committee (Experiments 1a and 2a), and by the Pediatric Clinic of the University of Padua (Experiments 1b and 2b). The individual depicted in the Fig 1 of this manuscript has given written informed consent (as outlined in PLOS consent form) to publish her photograph.

### Experiment 1a (4-month-old infants) Method

#### Participants

The final sample consisted of 20 healthy Japanese infants aged 4-months (14 male, 6 female, mean age = 122 days, ranging from 107 to 133 days). All participants are healthy full-term infants (birth weight ranging between 2480g to 3578g) with no known visual problem or developmental atypicality. An additional 6 infants were tested but were excluded from the analysis for not having at least one valid trial in each of the four test conditions.

#### Apparatus

All stimuli were displayed on a 24-inch wide LCD monitor (Dell S2409W) controlled by a computer. Tobii X120 which was connected to the same computer was placed just below the monitor to obtain eye-tracking data. The infant and the LCD monitor were located inside an enclosure, which was made of iron poles and covered with cloth. There were loudspeakers, one on either side of the CRT monitor. The experimenter could observe the infant behaviour live via a TV monitor connected to a digital camera positioned just below the monitor screen throughout the experiment.

#### Stimuli

Stimuli were two-frame dynamic images of three Asian females in full colour (see Fig 1a). There were three motion types: blinking, mouth opening, and nodding. Each dynamic stimulus consisted of two photograph of the same female face with a slightly different pose. The first frame of all dynamic stimuli was a face looking straight ahead with eyes open and mouth closed (see Fig 1a top). The second frame of the dynamic stimuli (see Fig 1a bottom) were either a face with closed eyes (blinking stimuli), a face with mouth open (mouth opening stimuli), or a face with her head tilted slightly down (nodding stimuli). The two frames in each dynamic stimulus were shown alternating with a duration of 500ms per frame. This produced the impression of blinking, mouth opening, or nodding. The dynamic stimuli always started with the first frame at the stimulus onset. Static stimuli were identical to the first frame of the dynamic stimuli. Each stimulus appeared in either its upright or inverted orientation. Each facial image subtended approximately 12°(547 pixel) × 15°(700 pixel) of visual angle (VA) and the distance between the nearest edges of the left/right image and central image was approximately 10° as viewed from a distance of approximately 50cm. All stimulus images were shown against mid-grey background (see Fig 1b).

Stimuli were paired so that facial orientation (upright vs. inverted) was manipulated between central and peripheral stimuli. There were four stimulus combination conditions as shown in Table 1. Each infant was shown 36 trials consisting of 4 stimulus combinations × 3 facial identities × 3 motion types in a random order within each experimental block. The left/right position of the peripheral stimuli on each trial was counterbalanced across 36 trials.

**Table 1:** Stimulus combination in Experiment 1a. All faces were dynamic as defined in the text.

Central face	Peripheral face
Upright	Upright
Upright	Inverted
Inverted	Upright
Inverted	Inverted

#### Procedure

Each infant sat on his/her parent’s or an experimenter’s lap in front of the LCD monitor. Before starting the experiment, calibration of the eye-tracker was performed using a 5-point calibration procedure for infants with Tobii Studio. Based on the calibration, eye-tracking data was obtained throughout the experiment and, using in-house program using Visual Basic 6.0 and Tobii Software Development Kit (SDK), was used to control stimulus presentation. Prior to the experiment, the area of the central stimulus and the two areas of the peripheral stimuli were defined. The eye-tracking data during each trail was also recorded and exported after the experiment.

Prior to each trial, an animated fixation point with a short beeping sound was presented at the center of the monitor. The experimenter pressed a key to initiate each trial as soon as the infant paid attention to the cartoon. The presentation of each trial was initiated only if the infant gaze tracking data was available and the infants gaze was located on the center area of interest (central AOI) at the timing of experimenter’s key press.

On each trial, a central stimulus appeared on the center of the LCD monitor for 2 seconds, followed by a peripheral stimulus either on the left or on the right to the central stimulus. After the onset of the peripheral stimulus, each trial terminated automatically if the infants gaze entered in the area of the peripheral stimulus (peripheral AOI). The experimenter terminated the trial if infants looked away from LCD monitor or looked away from the central stimulus, or if 5 seconds elapsed after the onset of the peripheral stimulus (the trial did not terminate automatically).

If infants were willing to attend to the monitor after the completion of the first block, the experimenter initiated one more block of stimulus presentation. Thus, each infant was allowed to receive as many trials until they complete two blocks of 36 trials. The experimenter terminated stimulus presentation at any stage of experiment if infants became inattentive to the monitor or fussy.

#### Data Analysis

The eye-tracking data collected at a rate of 60Hz during each trial. The eye-tracking data contained information about the x and y position of the infants gaze position at each time point. We defined AOIs corresponding to the central and peripheral stimulus position as shown in Fig 1b. Saccade latency was calculated by measuring the time from the onset of the peripheral stimuli to the time point when the infants’ gaze falls out of the central AOI. The central AOI corresponded to the area of central stimuli (12° × 15° area around the center of the monitor, see Fig 1b). The peripheral AOI corresponded to the area of peripheral stimuli (12° × 15° area, positioned 10° apart either from the left edge or the right edge of the central AOI, see Fig 1b).).

The saccade latency was analyzed from trials on which there was a well defined shift of gaze from the central toward the peripheral stimuli.

The saccadic latency was calculated by measuring the time from the onset of the peripheral stimuli to the time point when the infants’ gaze reached on the peripheral AOI. Total of 958 trials were observed across infants. Among these, trials were considered as invalid and rejected if (a) there was less than 500ms of continuous looking at the central stimuli immediately before the onset of the peripheral stimuli, or infants’ gaze was not on the central AOI (30%, 285 trials out of 958 trials), (b) the gaze shifted other than the peripheral stimulus direction (4%, 42 trials), (c) the gaze data was lost before infants’ gaze arrive in the peripheral AOI (17%, 164 trials), or (d) the saccade latency was less than 200ms (0%, 3 trials). Finally, based on previous studies (7, 9, 16), trials were discarded if (e) the gaze shift did not occur within 5sec of the onset of the peripheral stimulus (1%, 14 trials).

### Results

#### Number of valid trials

Table 2 shows the mean number of the valid trials and its proportion as a percentage among the number of trials for each condition shown to the infants.

**Table 2:** Mean (SD) number of the valid trials for each stimulus combination as a percentage of the total number of trials for each infant in Experiment 1a, 1b, 2a, and 2b.

	Central	Upright	Upright	Inverted	Inverted
	Peripheral	Upright	Inverted	Upright	Inverted
*Experiment 1a(4-months)*	*Number of valid trials*	6(4)	6(3)	5(3)	5(3)
*Experiment 1a(4-months)*	*%f valid trials*	47.59(24.03)	52.54(18.55)	44.71(25.56)	44.87(20.83)
*Experiment 1b(newborns)*	*Number of valid trials*	2(2)	3(2)	3(2)	3(1)
*Experiment 1b(newborns)*	*%f valid trials*	59.64(32.04)	59.64(25.71)	66.07(30.47)	63.1(25.68)
	Central stimuli	Static upright	Static inverted	Dynamic upright	Dynamic inverted
	Peripheral stimuli	Dynamic inverted	Dynamic upright	Static inverted	Static inverted
*Experiment 2a(4-months)*	*Number of valid trials*	5(4)	5(3)	6 (4)	4(4)
*Experiment 2a(4-months)*	*%f valid trials*	42.41(22.84)	47.04(19.35)	49.52(25.01)	42.84(20.15)
*Experiment 2b(newborns)*	*Number of valid trials*	3(1)	2(1)	2(1)	2 (1)
*Experiment 2b(newborns)*	*%f valid trials*	58.36(25.02)	43.83(18.1)	41.19(24.15)	49.93(21.19)

A 2 central stimulus orientation (upright/inverted) x 2 peripheral stimulus orientation (upright/inverted) repeated measures ANOVA on the proportion of valid trials revealed no significant effect or interaction (all *p’*s >.100).

#### Saccade latency

**Fig 2:**
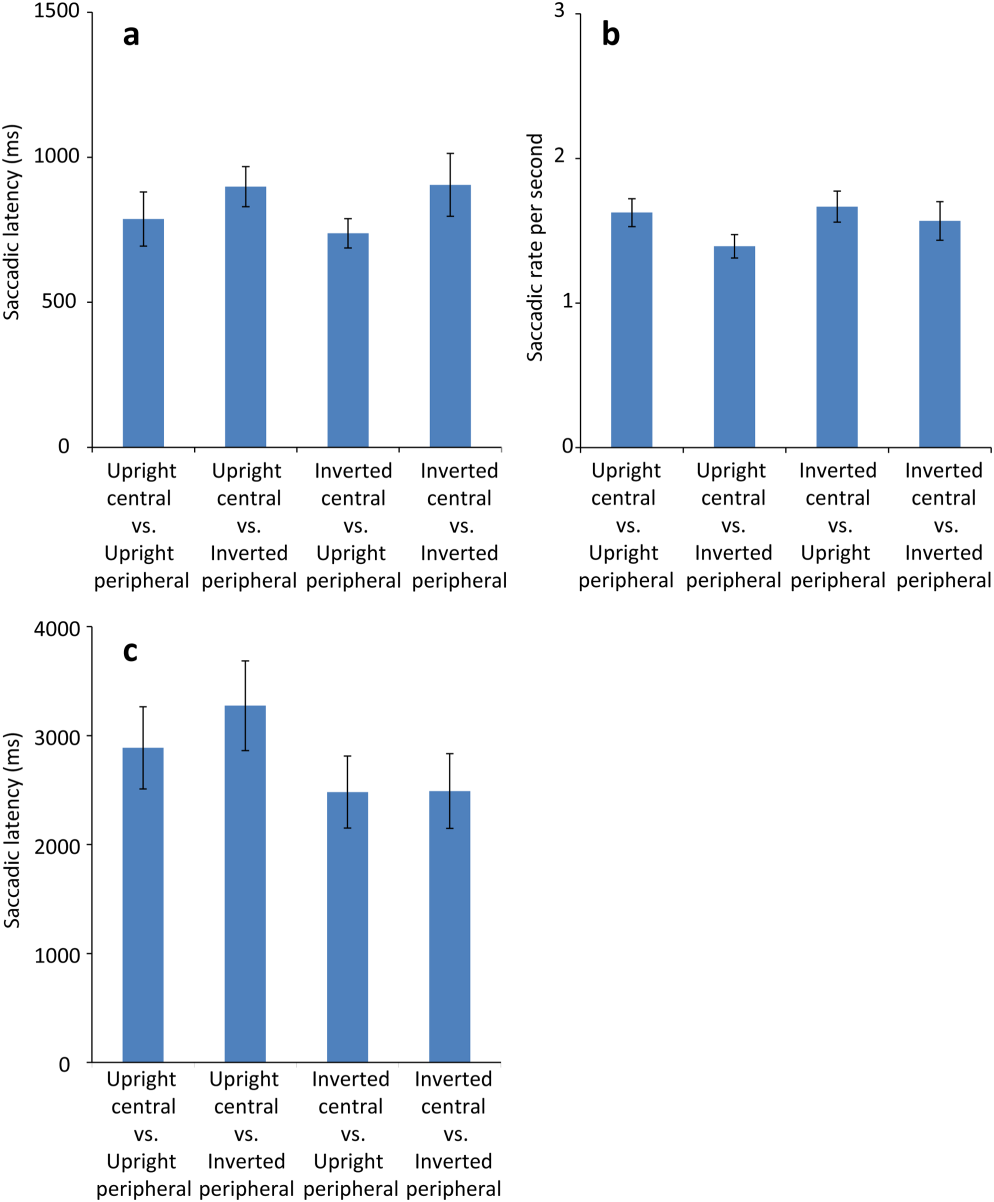
Results from Experiment 1. (a) Mean saccadic latency for each stimulus combination in 4month-olds (Experiment 1a). (b) Mean gaze saccadic rate per milliseconds in 4-month-olds (Experiment 1a). Note that greater values represent faster saccades. (c) Mean saccadic latency for each stimulus combination in newborn infants (Experiment 1b). Error bars in each graph represent +/-1 *SE*.

Fig 2a shows average saccadic latency in each stimulus combination condition. Shapiro-Wilk test with Bonferroni correction detected a significant deviation from normal distribution except for the inverted central vs. upright peripheral stimulus condition (all *p’*s < .050), due to data skew that typically occur for latency data. Therefore, we applied reciprocal transformation to latency data to compute the saccadic rate (Fig 2b). The saccadic rate was computed as 1000/latency for each trial. After transformation, visual inspection of histogram suggest skewness was reduced, and Shapiro-Wilk test with Bonferroni correction did not detect deviation from normal distribution for any of the saccadic rates (all *p’*s > .100). We performed a 2 central facial orientations (upright, inverted) x 2 peripheral face orientations (upright, inverted) repeated measures *ANOVA* on the saccadic rate. The analysis revealed a main effect of peripheral facial orientation alone, *F*(1,19) = 4.62, *p* = .045, *η*_*p*_^*2*^ *=* .20 (upright: *M* = 1.65 shift per seconds *SD* = 0.39; inverted: *M* = 1.48 shift per seconds, *SD* = 0.44). The greater saccadic rate for upright peripheral stimuli shows a faster gaze shift toward the upright stimuli regardless of the orientation of the central stimuli. No other effect or interaction reached significance (all *p’*s > .100).

### Experiment 1b (Newborn Infants) Method

#### Participants

The final sample consisted of fourteen 1- to 3-day-old Caucasian infants (*M* age = 38 h, *SD* = 17 h, range = 16–86 h). Nine additional newborns were tested but excluded from the final sample for not contributing at least one valid trial in each of the four test conditions. Infants had a normal delivery, birth weight between 2470 and 4050 g, and an Apgar score of 9 or 10 at 5 minutes. Newborns were tested only if awake and in an alert state. Parents (mother, father or both) were present during the observation of their son/daughter.

#### Apparatus

Stimuli were presented on a 30-inch Apple Cinema monitor 30 cm away from the newborn. Plain white curtains were drawn on both sides to prevent distraction. Above the monitor, a video camera (IVC 800) recorded the newborn to monitor the newborn’s looking behavior on-line and for off-line coding. The experimenter could observe the infant behaviour through a monitor connected to the video camera.

#### Stimuli

Stimuli were exactly the same utilized in Experiment 1a with 4-month-old infants (see Fig 1 and Table 1).

#### Procedure

Each newborn sat on an experimenter’s lap in front of the monitor at a distance of about 30 cm. This experimenter was naïve to the experimental hypothesis and blind regarding the stimuli presentation; he/she fixed his/her gaze on a camera on the ceiling throughout the session. A central flickering attention getter was used to attract the newborns’ attention at the start of the testing. When the newborn’s gaze was properly aligned with the attention getter, the sequence of trials was started by the experimenter who watched the newborn on a monitor and pressed a key on the computer keyboard which turned off the attention getter and activated the onset of the stimuli. As in Experiment 1a on each trial, a central stimulus appeared on the center of the monitor for 2 seconds, followed by a peripheral stimulus either on the left or on the right to the central stimuli. Also in Experiment 1b the distance between the nearest edges of the left/right image and central image peripheral stimulus was about 10° of visual angle (5.3 cm). The peripheral stimulus was presented until the newborn looked at it or until 10 seconds elapsed [8]. Once the newborn looked to the peripheral stimulus or if the maximum duration was reached, the experimenter pressed a key to replace the peripheral target with a central flickering attention getter and the next trial was presented.

Each newborn was shown 36 trials consisting of 4 stimulus combinations × 3 facial identities × 3 motion types divided into 3 blocks of 12 trials each. Within each block the 4 stimulus combinations were repeated in a random order 3 times each. The 3 facial identities and the 3 motion types were randomized along the entire experiment. The left/right position of the peripheral stimuli was randomized and counterbalanced across the 36 trials. If newborns were willing to attend to the monitor after the completion of the first block, the experimenter initiated the following block of stimulus presentation. Thus, each infant was allowed to receive up to two blocks of 36 trials. The experimenter terminated stimulus presentation at any stage of experiment if newborns became inattentive to the monitor or become fussy.

#### Data Analysis

In contrast to Experiment 1a where saccade latency was registered through a Tobii eye tracker system, in the present experiment video-recordings of the newborns’ looking behaviour were coded off-line frame by frame by two independent coders blind to the experimental hypothesis. As in previous experiment saccadic latency reflected the time taken by neonates to reach on the peripheral stimuli. Temporal resolution of the video recordings used to obtain newborn data was 25 frames per seconds.

A total of 248 trials were observed across all infants. Among these, trials were considered as invalid if (a) the newborn’s gaze was not on the central stimulus when the peripheral one appeared (11%), (b) the newborn did not look continuously at the central stimulus for at least 500ms immediately prior to the presentation of the peripheral stimulus (5%), (c) the newborn oriented towards the peripheral stimulus before 500ms (0%), (d) the newborn’s gaze did not shift directly to peripheral stimulus but moved in other directions before engaging the peripheral stimulus (9%), and (e) the newborn had their eyes closed for whatever reason (e.g. blinking, yawning; fussing) (14%). Finally, based on a previous study [8), trials were rejected if (f) the newborn did not orient towards the peripheral stimulus within 10000ms of its appearance (1%).

Inter-rater reliability calculated over 50% of the data was .9 (Cohen’s K) for the number of valid trials and correlation for saccadic latency was .87.

### Results

#### Number of valid trials

The mean number of the valid trials and its’ proportion in percentage among the number of trials for each condition shown to the newborns is shown in Table 2. A 2 central stimulus orientation (upright/inverted) x 2 peripheral stimulus orientaion (upright/inverted) repeated measures ANOVA on the proportion of valid trials revealed no significant effect of central stimulus orientation, *F*(1,13) = 0.46, *p* = .511, or peripheral orientation, *F*(1,13) = 0.07, *p* = .802, or their interaction *F*(1,13) = 0.12, *p* = .739, suggesting that newborns’ proportion of valid trial was similar for all the conditions.

#### Saccade latency

Fig 1c shows average saccadic latency in each stimulus combination condition. Shapiro-Wilk test with Bonferroni correction detected no significant deviation from normal distribution (*p*s > .1). As in the previous experiment we performed a 2 central facial orientations (upright, inverted) x 2 peripheral face orientations (upright, inverted) repeated measures *ANOVA* on saccadic latency. The analysis did not reveal any main effect of central facial orientation, *F*(1,13) = 2.66, *p* = .127, or of peripheral facial orientation *F*(1,13) = 0.26, *p* = .619, or of their interaction *F*(1,13) = 0.24, *p* = .636.

### Discussion of Experiment 1

Taken together the results of Experiments 1 revealed that an influence of face orientation on the deployment of visual attention occurred at 4 months but not at birth. In 4-month-old infants, the number of valid trials was also greater when the central stimulus was upright face compared to when it was inverted face (see Table 2). Considering that most of the trial rejection was due to inattention to the central stimuli before or at the peripheral stimulus onset, the results suggest that upright central stimuli are more effective in holding infants’ attention than the inverted stimuli. Further, their orienting occurred more quickly when the peripheral stimulus was an upright face compared to when it was an inverted face (Experiment 1a). Unlike in the current study, Hunnius and Geuze [9] reported that 10 to 18 weeks old infants shift their gaze more frequently when the central stimulus was a dynamic face and the peripheral target was a dynamic abstract stimulus. As they used mother’s face as the face stimuli, Hunnius and Geuze discussed that their results suggest that mother’s face being less able to hold or attract the attention of the infants. Such a view would explain the difference between the results of Hunnius and Geuze and the results from 4-months in the current study. Unlike the special case of mother’s face, the current results obtained with unfamiliar faces suggest that upright faces in general hold and attract attention of infants around 4-months of age.

In contrast to 4-month-olds, newborns did not show any effects of face orientation on the proportion of valid trials or on saccade latency (Experiment 1b). The finding with newborns are consistent with Farroni et al’s [8] study, in which saccade latency for upright face-like target were no faster than for inverted ones in the overlapping condition.

Overall the results of Experiments 1 suggest that the face orientation differently affects orienting of visual attention at 4 months of age than at birth. Most probably, at birth attention is strongly triggered by the salience of a general attribute of the stimulus such as motion, and consequently the face orientation is not sufficient to affect orienting unlike four months later. To test this explanation, a second experiment was carried out in which motion and face orientation were directly contrasted.

## Experiment 2

To better understand the results of Experiment 1, a second Experiment was carried out in which we varied the face orientation and motion of the central and peripheral stimuli. We expected face orientation to be the main factor affecting the orienting of visual attention at 4 months, but motion to be more influential at birth.

### Experiment 2a (4-month-old infants) Method

#### Participants

The final sample consisted of 18 healthy Japanese infants aged 4-months (9 male, 9 female, mean age = 120 days, ranging from 111 to 133 days). All participants were healthy, full-term infants (birth weight ranging between 2476g to 3500g) with no known visual problem or developmental atypicality. An additional 4 infants were tested but were excluded from the analysis for not contributing at least one valid trial in each of the four test conditions.

#### Apparatus, Stimuli. Procedure, and Data Analysis

Apparatus, Stimuli. Procedure, and Data Analysis were same as those in Experiment 1 except for the following. Face motion as well as orientation was contrasted between the central and peripheral stimuli. Static stimuli were created by taking the first frame of the dynamic stimuli used in Experiment 1 (see Fig 1) for each motion condition and face. This gave the four stimulus combination conditions listed in Table 3.

**Table 3:** Stimulus combination in Experiment 2.

Central face	Peripheral face
Dynamic Upright	Static Inverted
Dynamic Inverted	Static Upright
Static Upright	Dynamic Inverted
Static Inverted	Dynamic Upright

A total of 764 trials were observed across infants. Among these, trials were considered as invalid if (a) there was less than 500ms of continuous looking at the central stimuli immediately before the onset of the peripheral stimuli, or infants’ gaze was not on the central AOI (31.94%, 244 trials), (c) the gaze shifted other than the peripheral stimulus direction (5%, 39 trials), (d) the gaze data was lost before infants shift gaze toward the peripheral stimuli (11%, 109 trials), or (e) the saccade latency was less than 200ms (0%, 4 trials). Finally, based on previous studies [7, 9, 16], trials were discarded if (f) the gaze shift did not occur within 5sec after onset of the peripheral stimulus (1%, 7 trials).

### Results

#### Number of valid trials

The mean number of the valid trials and its proportion as a percentage of the number of trials for each condition shown to the infants is shown in Table 2.

A two-way *ANOVA* with 2 levels of movement property (dynamic central + static peripheral, static central + dynamic peripheral) x 2 levels of stimulus orientations (upright central + inverted peripheral, inverted central + upright peripheral) on the proportion of valid trials revealed a marginally significant interaction, *F*(1,17) = 3.53, *p* = .08, *η*_p_^2^ = .17. Simple effect analysis following up the interaction revealed no significant effect (*p* < .010).

#### Saccade latency

**Fig 3:**
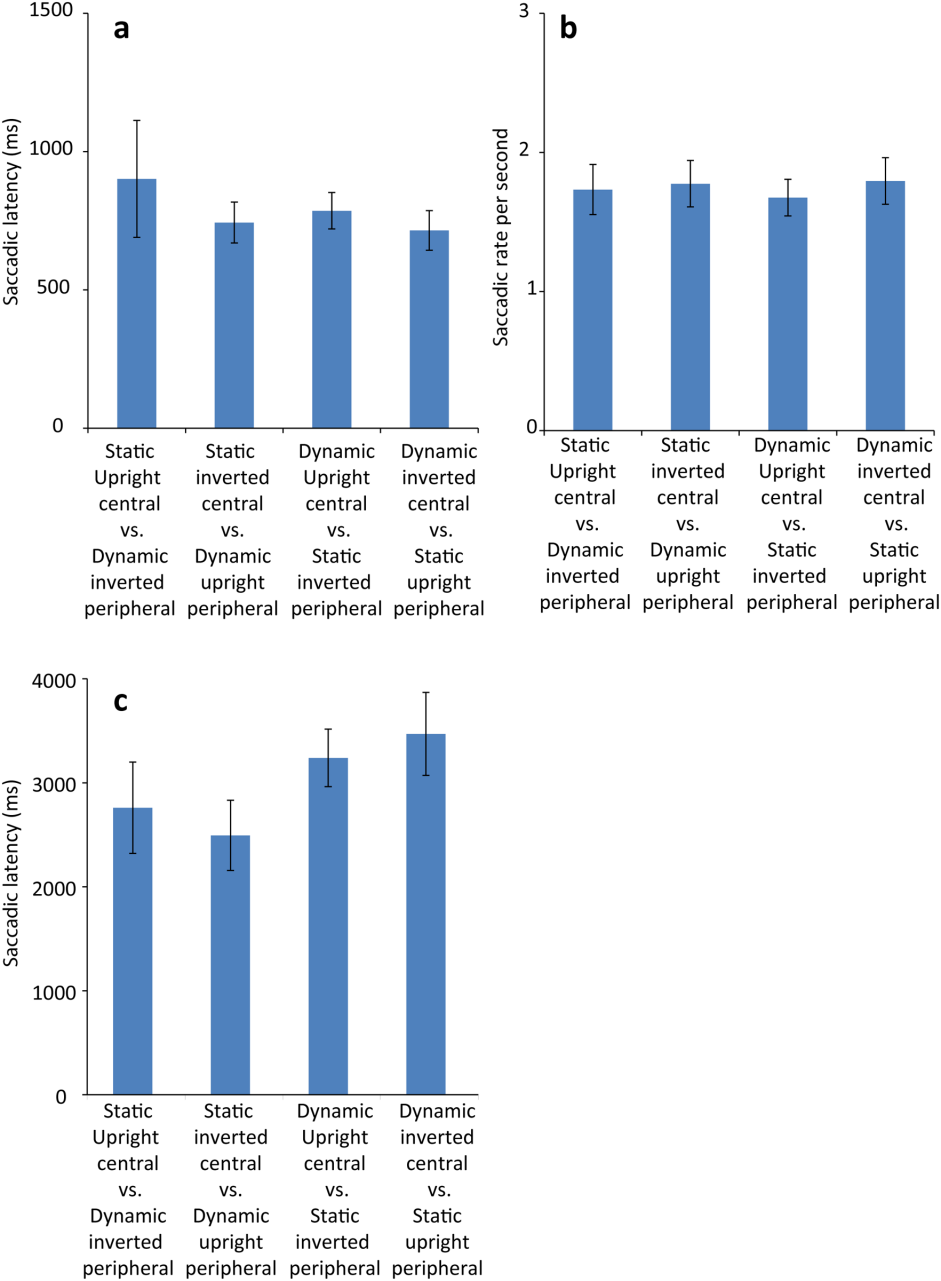
Results from Experiment 2. (a) Mean saccadic latency for each stimulus combination in 4month-olds (Experiment 2a). (b) Mean saccadic rate per second in 4-month-olds (Experiment 2a). Note that greater values represent faster saccades. (c) Mean saccadic latency for each stimulus combination in newborns infants (Experiment 2). Error bars in each graph represent +/-1 *SE*.

Fig 3a shows average saccadic latency in each stimulus combination condition. Shapiro-Wilk test with Bonferroni correction detected a significant deviation from normal in static upright central vs. dynamic inverted peripheral stimulus condition (*p* < .010), due to the positive data skew typical of latency data. Therefore, we applied the reciprocal transformation to the saccadic latency data to give saccadic rate (Fig 3b) as in Experiment 1. After transformation, visual inspection of histogram suggest skewness was reduced, and Shapiro-Wilk test with Bonferroni correction did not detect deviation from normal distribution for any of the gaze shift rates (*p’*s > .100). We performed a two-way repeated *ANOVA* with 2 levels of movement property (dynamic central + static peripheral, static central + dynamic peripheral) x 2 levels of stimulus orientation (upright central + inverted peripheral, inverted central + upright peripheral) on the saccadic rate. The analysis revealed no significant main effect or interaction (all *p’*s > .100). While Fig 3b shows a trends of greater saccadic rate for the conditions with upright peripheral stimuli (which is consistent with a trends of a shorter saccadic latency for these conditions as seen in Fig 3a), it did not reach significance.

### Experiment 2b (newborn infants) Method

#### Participants

The final sample consisted of 14 healthy 1- to 3-day-old newborns (*M* age = 40 h, *SD* = 15 h, range = 20–92 h). An additional 3 newborns were tested but were excluded from the analysis for not contributing at least one valid trial in each of the four test conditions. Infants had a normal delivery, birth weight between 2470 and 4050 g, and an Apgar score of 9 or 10 at 5 minutes. Newborns were tested only if in an awake and in alert state.

#### Stimuli

Stimuli were exactly the same as for Experiment 2a (see Table 3).

#### Apparatus, Procedure, and Data Analysis

Apparatus, Procedure, and Data Analysis were same as for Experiment 1b, except for the following. A total of 252 trials were observed across newborns. Among these, trials were considered as invalid if (a) the newborn’s gaze was not on the central stimulus when the peripheral one appeared (14%), (b) the newborn did not look continuously at the central stimulus for at least 500ms immediately prior to the presentation of the peripheral stimulus (3%), (c) the newborn oriented towards the peripheral stimulus before 500ms (0%), (d) the newborn’s gaze did not shift directly to the peripheral stimulus but moved in other directions first (10%), (e) the newborn had their eyes closed for whatever reasons (e.g. blinking, yawning; fussing) (16%). Finally, based on previous work [8], trials were also considered as invalid if (f) the infant only oriented towards the peripheral stimulus more than 10000ms of its appearance (5%).

### Results

#### Number of valid trials

Table 2 shows the mean number of valid trials and its proportion as a percentage of the total the number of trials for each condition shown to the newborns.

A two-way ANOVA with 2 levels of movement property (dynamic central + static peripheral, static central + dynamic peripheral) x 2 levels of stimulus orientation (upright central + inverted peripheral, inverted central + upright peripheral) on the proportion of valid trials revealed a significant interaction between movement property and stimulus orientation *F*(1,13) = 5.03, *p* = .043, *η*_p_^2^ = 0.28. Simple effect analysis showed a marginally significant effect of movement property only for the combination of upright central vs inverted peripheral, *t*(13) = 2.08, *p* = .058. Newborns showed a higher proportion of valid trials with static central upright face than dynamic central upright face, suggesting that the stimulus attribute that influence their proportion of valid trials is different by those that affected 4-month-old infants.

#### Saccade latency

Fig 3c shows average saccadic latency for each stimulus combination condition. Shapiro-Wilk test with Bonferroni correction detected no significant deviation from normal (p > .080 for the upright static/inverted dynamic condition and *p*s > .100 for the other 3 conditions). A two-way *ANOVA* with 2 levels of movement properties (dynamic central + static peripheral, static central + dynamic peripheral) x 2 levels of stimulus orientations (upright central + inverted peripheral, inverted central + upright peripheral) on the saccadic latency revealed a significant main effect of dynamic properties, *F*(1,13) = 7.80, p = .015 *η*_p_^2^ = 0.38, with longer latencies in the dynamic central condition (*M* = 3354 ms, *SD* = 237 ms) than in the static central condition (*M* = 2626, *SD* = 333 ms). The main effect of stimulus orientation, *F*(1,13) = 0.01, *p* = .948, and the interaction between the two factors, *F*(1,13) = 0.46, *p* = .508, were not significant

### Discussion of Experiment 2

Unlike our prediction, neither the dynamic property nor the facial orientation influenced dominantly to the orienting behaviour in 4-month-olds. On the other hand, both the proportion of valid trials and the saccadic latency showed that the dynamic property predominantly influenced to the orienting behaviour in newborns.

## General Discussion

In the present study we investigated the role played by face orientation and motion on orienting visual attention in 4-month-old infants and newborns. Findings showed that these stimulus attributes differently modulate the orienting of attention between 4-month-old infants and newborns, revealing developmental change during the first months of life.

A first difference between 4-month-old infants and newborns concerns the saccadic latency to the peripheral target: Four-month-old infants are faster than newborns in shifting their gaze toward a target in the periphery when a stimulus persists in the center of the screen. Our results support previous findings that show a substantial decrease of saccadic latency between 9 and 16 weeks [5, 9, 15, 16], a timing that coincides with a period of increased activity in the cortical area thought to mediate the disengagement and shifting of attention and gaze [12, 42]. The difference in speed of orienting between 4-month-old infants and newborns may be explained as the consequence of the maturation of the cortical area that mediate the orienting of visual attention [12, 56].

A second difference between 4-month-old infants and newborns concerns the pattern of attentional behaviour. Overall both Experiments 1 and 2 revealed that the orienting of attention is mainly influenced by motion but not by face orientation at birth, but it is also influenced by face orientation at 4-months of age. The proportion of valid trials in newborns was higher when the central stimulus was a static upright face (Experiment 2b), while it was the same regardless of the upright and inverted orientation of faces when both the central and the peripheral stimulus were dynamic (Experiment 1b). Similarly newborns’ saccadic latency was not influenced by facial orientation when both the central and the peripheral stimuli were dynamic (Experiment 1b). Again, irrespective of the orientation of the face, newborns’ saccadic latency was slower when the central stimulus was dynamic and the peripheral stimulus was static compared to the opposite (Experiment 2b). By contrast, Experiment 1 and 2 suggest that both facial orientation and motion influence the orienting of attention in 4-month-olds. Four-month-old infants’ saccades occurred more quickly when the peripheral stimulus was an upright as compared to an inverted face when both the central and the peripheral stimulus were in motion (Experiment 1a). However, the influence of the facial orientation disappeared when the face orientation attribute was contrasted with the dynamic attribute (Experiment 2a).

A limitation of this study is that we are not able to disentangle which stimulus (i.e., the central one ore the peripheral one) was more responsible for the observed effects. For example, any latency effect found in the current study could have been due to an increase in shifting speed to moving peripheral stimuli or, conversely, slowed disengagement from moving central stimuli. Future research that involves greater number of conditions would be needed to establish which one was the case.

Why would 4-month-old infants be influenced by a different stimulus attribute compared to newborn infants? There are at least three possible interpretations of our finding. First, our findings may partly reflect an unequal sample sizes between the age groups. Indeed, both 4-month-old experiments report findings on 20 infants, whereas both newborn experiments report findings on 14 newborns. Given that in Experiment 1b we obtained a null result, the difference in statistical power may explain for the different pattern of results. However a significant statistical effect was obtained in Experiment 2b with the same sample size for newborns. Moreover our findings for newborns are consistent with Farroni et al’s [8] study, in which saccade latency was no faster for upright face-like target than for inverted ones in the overlapping condition. Second, data were collected in two different labs with infants drawn from different populations. While exactly the same procedure and stimuli were employed, a different testing apparatus was utilized. Indeed saccadic latency was registered through a Tobii eye tracker system in the experiments with 4-month-old infants but coded offline from video-recordings of looking behaviour for newborns. While the use of an eye tracker may result in a more accurate and objective recording of saccadic latency than laboratory observation, it should not influence the pattern of attentional behaviour shown by participants. Instead, we conclude that the change in the pattern of attentional behaviour from birth to 4 months is not artefactual but real, and that our results fit the early and rapid development of face processing in the first year of life [36, 57–65] that mainly result from postnatal face experience [63, 66–70]. As outlined in the introduction, stimulus properties that elicit face preference undergo considerable developmental change in the first few months of life. Newborns, with their minimal experience with faces, prefer not only faces but also non-face like patterns that share certain structural properties with faces, i.e. top-heavy properties [33, 65] or congruency [71], suggesting that they possess facial representation in which only very general structural properties are embedded. For these infants, movement, which easily triggers attention from birth [18, 19], may compete with attention to upright faces, hence weakening the newborn’s attentional orientating toward faces. Such competitive rather than facilitative effect of motion information on newborn’s attention to face would account for the lack of any advantage for upright faces in Experiment 1. Consistent with this, the results from Experiment 2 demonstrated that motion information dominates face orientation in eliciting an orienting response from newborns.

By 3-months of age, infants develop more specific representations of facial configuration, with more realistic facial characteristics required to elicit face preference in infants [35]. Perhaps because they encounter faces nearly exclusively in motion during their daily interactions, motion information is also shown to facilitate perception and attention to faces in infants by around 4-months of age [72–74]. In line with these previous findings our results suggest that movement of a real face elicits and supports attention and rapid orienting towards faces in 4-month-old infants unless the movements are in competition with face orientation. Overall, we believe that the current report has demonstrated that face orientation and motion differently affect the deployment of visual attention in newborns and 4-month-old infants

Visual experience with faces is anyway not the only opportunity that infants have to learn about people. Indeed the infants are not passive observers and their motor experience and emerging exploration skills increase their spontaneous interest in and attention towards faces [75–76]. For example, self-produced reaching experience strengthens a spontaneous preference for faces over objects in 3-month-old infants and facilitates infants’ perception and understanding of observed action [75]. Moreover infants’ motor activity level, assessed via parent report, predicts face preference in 3-month-old infants [76]. These findings suggest that new motor skills provide infants with different ways to interact with others, and alter their attention to and engagement with social interaction partners. Given that motor ability advances considerably during the first 4 months of life, it creates opportunities for infants to interact with objects and people in new and progressively more sophisticated ways. This, in turn, increases motor and social stimulations, and impose infants with an increased need to dynamically shift and guide their attention. At the same time, increased motor and social stimulations may also boost their social development. Interestingly, a study in mice have shown that an enriched environment that provide pups with more opportunities for motor activity and social exchanges accelerates the development of visual system [77]. Considering that the development of motor skills in infants is associated with increased motor and social stimulations, it would act to enrich social environment. Consequently, it may act to accelerate social development. When considered together, not only passive experience with faces, but also the development of motor skills and the resultant active engagement with others would jointly account for the currently observed developmental change in the orienting behaviour in the first 4-months of life.

## Supporting Information

S1 Data Raw data of the percentage of valid trials for both the 4-month-olds’ experiments (Experiment 1a and 2a) and the newborns’ experiments (Experiments 1b and 2b). (XLSX)

S2 Data Raw data of the saccadic latencies for the 4-month-olds’ experiments (Experiments 1a and 2a) and the newborns’ experiments (Experiments 1b and 2b). (XLSX)
